# Effects of Traditional Moroccan *Bandek* Processing on Nutritional Composition, In Vitro Starch Digestibility, and Volatile Profiles of Barley and Durum Wheat Genotypes

**DOI:** 10.3390/foods15183250

**Published:** 2026-09-14

**Authors:** Kubra Ozkan, Lorenzo Palombi, Abderrazek Jilal, Ghizlane Salih, Francesco Sestili, Cagla Ozer, Maria Tufariello, Barbara Laddomada, Osman Sagdic, Andrea Visioni, Samuela Palombieri, Hamit Koksel

**Affiliations:** 1 Department of Food Engineering, Faculty of Chemical and Metallurgical Engineering, Davutpasa Campus, Yildiz Technical University, Istanbul 34349, Türkiye; kubraozkan1907@gmail.com (K.O.); osagdic@yildiz.edu.tr (O.S.); 2 Institute for Applied Physics “Nello Carrara” (IFAC), National Research Council (CNR), 50019 Firenze, Italy; l.palombi@ifac.cnr.it; 3 National Institute for Agricultural Research of Morocco (INRAM), Rabat P.O. Box 415, Morocco; abderrazek.jilal@inra.ma (A.J.); ghizlane.salih@inra.ma (G.S.); 4 Department of Agriculture and Forest Sciences (DAFNE), University of Tuscia, 01100 Viterbo, Italy; francescosestili@unitus.it; 5 Department of Gastronomy and Culinary Arts, Faculty of Fine Arts, Design and Architecture, Istinye University, Istanbul 34010, Türkiye; cozer@istinye.edu.tr; 6 Institute of Sciences of Food Production (ISPA), National Research Council (CNR), Via Monteroni, 73100 Lecce, Italy; maria.tufariello@cnr.it; 7 Institute of Sciences of Food Production (ISPA), National Research Council (CNR), Via Amendola 122/O, 70126 Bari, Italy; barbara.laddomada@cnr.it; 8 Biodiversity and Crop Improvement Program, International Center for Agricultural Research in the Dry Areas (ICARDA), Rabat P.O. Box 415, Morocco; a.visioni@cgiar.org

**Keywords:** traditional cereal processing, β-glucan, resistant starch, glycemic index, volatile organic compounds

## Abstract

Traditional processing can modify the nutritional and volatile characteristics of cereal-based foods. This study evaluated Moroccan *Bandek* production, comprising soaking, steaming, solar drying, and roasting, using three durum wheat genotypes—conventional Svevo, high-amylose Svevo HA, and soft-kernel Faridur—and the high-β-glucan hull-less barley Chifaa. Raw wholemeal flours and corresponding *Bandek* products were analyzed for color, protein, β-glucan, resistant starch, iron, zinc, phenolic compounds, antioxidant capacity, and volatile organic compounds (VOCs). In vitro starch hydrolysis and predicted glycemic index (pGI) were determined in *Bandek* products. Compared with the corresponding flours, *Bandek* products were darker and showed higher resistant starch, extractable phenolic compounds, antioxidant capacity, iron, and zinc concentrations, whereas protein content was lower. However, marked differences emerged among the cereal matrices. Among the durum wheats, Svevo HA *Bandek* had the highest resistant starch content (6.82%) and a lower pGI than conventional Svevo, whereas Faridur showed the highest pGI (74.75%). In contrast, Chifaa barley *Bandek* retained 6.03% β-glucan and had the lowest pGI (53.78), clearly distinguishing it from the durum wheat products. Following standardized sample preparation, total semi-quantitative VOC abundance was higher in *Bandek* than in flour, with pyrazines providing the clearest distinction between the processed and raw matrices and additional variation attributable to genotype. These findings demonstrate that *Bandek* characteristics depend strongly on the cereal species and genotype, highlighting the distinct nutritional potential of high-β-glucan barley and high-amylose durum wheat.

## 1. Introduction

Traditional cereal processing methods constitute complex transformation systems that significantly influence the nutritional and functional properties of cereal-based foods. Techniques such as soaking, steaming, fermentation, roasting, and malting have long been used across African and Middle Eastern food systems not only to improve preservation, edibility, and sensory quality, but also to induce structural and biochemical changes that affect nutrient bioavailability, starch digestibility, and the technological performance of cereal flours [1]. In traditional African contexts, cereal pre-treatments, including steeping, parboiling, tempering, and roasting, play a central role in shaping product structure and sensory acceptability, although they are often not standardized [2]. Several of these treatments can also reduce antinutritional factors, particularly phytates, thereby improving the bioavailability of essential minerals such as iron and zinc [3]. Fermentation and malting, for instance, activate endogenous phytases, leading to phytic acid degradation and enhanced functional properties. Thermal processing represents a key driver of biochemical transformations in cereal matrices. Operations such as steaming, drying, and roasting induce molecular changes that affect phenolic composition, antioxidant capacity, volatile profile, starch structure, and glycemic behavior [4]. In cereals, phenolic compounds are predominantly present as bound forms associated with cell wall components, limiting their extractability in raw grains [5,6]. Thermal treatments can disrupt these structures, promote the release of bound phenolics and increase antioxidant activity [4,7]. Concurrently, hydrothermal processing and roasting modify starch organization, influencing the balance between rapidly digestible, slowly digestible, and resistant starch fractions, with implications for postprandial glycemic response [8,9]. Roasting further promotes Maillard reactions, contributing to color development, flavor formation, and potentially enhanced antioxidant properties.

At the molecular level, sequential hydrothermal and thermal treatments may induce some of the physicochemical transformations observed during extrusion, although without the intense pressure and shear forces involved in the latter. Soaking facilitates water penetration into the endosperm, while subsequent steaming promotes starch gelatinization and protein unfolding. Drying and cooling can favour starch-chain reassociation and RS3 formation, whereas roasting may further induce protein aggregation and starch–protein or starch–lipid interactions. The extent of these transformations depends on both processing conditions and the intrinsic composition of the cereal genotype [10,11,12,13]. These transformations are not expected to occur uniformly across the processing sequence: resistant starch formation is likely driven mainly by the steaming–cooling/drying stages, whereas roasting is expected to dominate Maillard-derived browning and pyrazine formation and may also modify phenolic extractability and antioxidant measurements. Within this context, roasted cereal flours occupy a prominent place in Moroccan culinary heritage. Traditional products such as *sellou* (sfouf) and *zemita* are emblematic preparations derived from roasted barley or wheat flour, often combined with sesame seeds, nuts, sugar, and fat [14,15]. These foods are deeply embedded in social practices, particularly during religious and festive occasions, and reflect longstanding traditions of cereal transformation [16]. Beyond their nutritional role, such preparations embody intergenerational knowledge transfer, gendered culinary practices, and collective food processing rituals that reinforce cultural identity [17]. However, ongoing urbanization has led to simplification of these multi-step procedures, which are increasingly replaced by the direct roasting of refined flours. This shift underscores the need for scientific documentation and characterization of artisanal cereal-processing systems, both to advance food science and to preserve intangible food heritage.

Although individual processing techniques and industrial treatments have been extensively investigated, traditional multi-step artisanal systems remain comparatively understudied. In these systems, sequential operations may produce cumulative or interactive effects that cannot be inferred from the evaluation of each processing step in isolation. This knowledge gap is particularly evident for North African cereal-based foods. Moreover, few studies have compared cereal genotypes with contrasting compositions under identical artisanal processing conditions, limiting current understanding of genotype × processing interactions.

Recent advances in cereal breeding have enabled the development of wheat and barley genotypes specifically enriched in health-promoting components, including resistant starch and β-glucans [18,19]. Although these traits offer promising opportunities for developing foods with improved nutritional profiles, their retention and functionality may be strongly affected by processing. It is therefore necessary to determine whether traditional artisanal treatments preserve, enhance, or diminish these beneficial characteristics and how the response varies according to the cereal matrix.

Against this background, the present study investigated *Bandek*, a traditional Moroccan roasted cereal flour obtained through sequential steaming, solar drying, and direct-fire roasting and used in the preparation of products such as *sellou* and *zemita*. Despite its cultural and nutritional importance, *Bandek* remains poorly documented in the scientific literature and has not yet been comprehensively characterized.

Four cereal genotypes with contrasting technological and nutritional characteristics were specifically selected to assess how the intrinsic properties of the raw material interact with traditional *Bandek* processing. The conventional durum wheat cultivar Svevo was included as a reference genotype representative of standard durum wheat quality. Its high-amylose derivative, Svevo HA, was selected for its enhanced nutritional value, particularly its naturally high resistant starch content, and to determine whether the hydrothermal treatments and subsequent cooling involved in *Bandek* production could further increase resistant starch formation through starch retrogradation. The hull-less barley cultivar Chifaa was included because of its high dietary fiber content, particularly β-glucans, which may improve the nutritional and glycemic properties of the final product. Finally, the soft-kernel durum wheat cultivar Faridur was selected to investigate the influence of reduced kernel hardness and distinctive milling behavior while retaining the quality attributes and characteristic flavor of durum wheat.

These contrasting characteristics were expected to influence starch gelatinization and retrogradation, water absorption and retention, milling behavior, dietary fiber functionality, and the availability of precursors involved in thermal and aroma-forming reactions. Accordingly, the study evaluated the effects of traditional processing on the nutritional, physicochemical, functional, volatilomic, and predicted glycemic properties of the four cereal matrices. The *Bandek* samples were produced by experienced local processors in the Tata region of southern Morocco, following the traditional artisanal practices of the Tata oasis. By integrating nutritionally improved cereal germplasm with a culturally embedded processing practice, this work provides new insights into the effects of both genotype and traditional processing and their interaction on the quality of cereal-based foods. At the same time, it contributes to the scientific valorization of Moroccan food heritage and supports the development of sustainable functional foods rooted in local knowledge and practices.

## 2. Materials and Methods

### 2.1. Plant Material

Three durum wheat (*Triticum turgidum* ssp. *durum*) genotypes—(i) cv. Svevo (with normal amylose content), (ii) high-amylose Svevo (Svevo HA), (iii) soft-kernel durum cv. Faridur, and the high-β-glucan hull-less barley (*Hordeum vulgare* L.) cv. Chifaa—were used in this study as raw materials for *Bandek* production. Svevo is a widely cultivated Italian durum wheat cultivar commonly used as a reference genotype in cereal quality and functional studies because of its stable agronomic performance and technological aptitude [20]. Faridur is the first Italian soft kernel durum wheat variety developed through the introgression of puroindoline genes into durum wheat, resulting in reduced kernel hardness and modified milling properties compared with conventional durum wheat cultivars [21]. Svevo HA is a high-amylose durum wheat genotype characterized by increased resistant starch accumulation and altered starch composition due to modifications in starch biosynthesis pathways, previously described for its enhanced nutritional and functional properties [18,22].

Chifaa is a hull-less barley variety characterized by high β-glucan content and recognized for its potential health-promoting properties, particularly in relation to glycemic response modulation and dietary fiber enrichment [19]. The inclusion of these cereal genotypes allowed the evaluation of the interaction between genotype composition and technological processing on the nutritional and functional quality of *Bandek*.

### 2.2. Bandek Production

*Bandek* was produced in the Tata region of southern Morocco according to the traditional procedure used by local processors. Mature cereal grains were processed through sequential cleaning, soaking, steaming, solar drying, roasting, cooling, and milling. Each sample (approximately 1 kg) was processed separately as a single batch to prevent cross-contamination. All samples were processed during the same production period and under comparable conditions. Grains were manually cleaned to remove dust, stones, broken kernels, chaff, and other foreign materials. The cleaned grains were immersed in potable water at a grain-to-water ratio of 1:3 (*w*/*v*) and soaked overnight for 12 h. After soaking, excess water was drained before steaming. The hydrated grains were steamed for 45–60 min using traditional steaming equipment. Steaming was continued until the kernels were fully hydrated and softened while retaining their structural integrity. After steaming, the grains were spread in uniform layers approximately 1 cm thick and solar-dried under the environmental conditions of the Tata region, characterized by intense solar radiation, low relative humidity, and high daytime temperatures. During drying, ambient temperatures ranged from 28 to 36 °C. The grains were covered overnight to limit moisture reabsorption while maintaining air circulation, and solar exposure was resumed during the daytime. Solar drying was terminated according to the traditional processing procedure. Moisture analysis of the samples was performed according to the American Association of Cereal Chemists (AACC) Method 44-15A [23]. Each dried grain sample was roasted separately according to a consistent operational protocol. To minimize processing variability, all samples were roasted during the same production period by the same experienced local processor, using the same traditional metal pan and wood-fire configuration. The grains were continuously stirred throughout roasting to ensure uniform heat transfer and prevent localized scorching. Roasting lasted 35–50 min, with the exact duration adjusted to achieve a predefined traditional endpoint, consisting of a uniform golden-brown color, a characteristic toasted aroma, and a crisp kernel texture without visible scorching. The same batch size, equipment, operator, stirring procedure, and endpoint criteria were applied to all cereal genotypes. Although pan-surface temperature was not instrumentally recorded, these controlled operational conditions ensured that all samples were subjected to a comparable and reproducible artisanal roasting procedure.

After roasting, the grains were spread in a thin layer and allowed to cool naturally to ambient temperature before milling. The roasted grains were milled using a small-scale industrial six-roller mill powered by a 27-hp motor. The resulting flour was screened using a manual plansifter solely to remove coarse particles that had not been sufficiently reduced during milling. No fractionation or bran separation was performed. The final wholemeal roasted flour, with an extraction rate of approximately 100% and a mean particle size of approximately 500 μm, was immediately transferred to airtight, food-grade containers and stored at room temperature under cool and dry conditions until physicochemical, nutritional, functional, and volatile-compound analyses were performed. Thus, all processing steps were performed by experienced local processors according to a consistent operational protocol that preserved the authentic artisanal practices traditionally used for *Bandek* production in the Tata oasis. For each genotype, all subsequent measurements were analytical replicates obtained from the same *Bandek* batch.

### 2.3. Physicochemical, Nutritional, and Mineral Analyses

Physicochemical, nutritional, and mineral analyses were carried out on wholemeal flours produced from untreated grains of the three durum wheat genotypes (Svevo, Svevo HA, and Faridur) and the hull-less barley cultivar Chifaa, together with the corresponding *Bandek* products obtained from the same cereal genotypes. To ensure comparability, the unprocessed grains and their corresponding *Bandek* samples were milled separately using the same small-scale industrial six-roller mill driven by a 27-hp motor. All milled fractions were retained and combined, with no bran removal or compositional fractionation, resulting in wholemeal flours with an extraction rate of approximately 100%. The total nitrogen content was determined according to AACC Method 46–10.01 [24], and protein content was calculated using a conversion factor of 6.25 (protein = *N* × 6.25).

The β-glucan content was determined enzymatically using the Megazyme β-Glucan Assay Kit (Megazyme International Ireland Ltd., Wicklow, Ireland), following the manufacturer’s instructions.

Resistant starch (RS) content was quantified according to AOAC Method 2002.02 [25] and AACCI Method 32–40.01 [26] using a Resistant Starch Assay Kit coupled with a glucose assay kit (Megazyme International, Wicklow, Ireland).

Color parameters were evaluated in the CIELAB color space using a Konica Minolta CR-400 colorimeter (Konica Minolta, Tokyo, Japan). The color coordinates recorded were L* (lightness), a* (redness/greenness), and b* (yellowness/blueness). In addition, the Browning Index (*BI*), used as an indicator of browning intensity induced by thermal processing, was calculated according to the following equations:

(1) $$BI=\frac{100(x-0.31)}{0.172}$$

where

(2) $$x=\frac{a^{*}+1.75L^{*}}{5.645L^{*}+a^{*}-3.012b^{*}}$$

Mineral composition, specifically iron (Fe) and zinc (Zn) contents, was determined by Inductively Coupled Plasma Optical Emission Spectroscopy (ICP-OES), following the procedure described by Palombieri et al. [27]. Results were expressed as ppm.

### 2.4. Determination of Phenolic Contents and Antioxidant Capacities

Phenolic compounds (free and bound) of the flour and *Bandek* samples were extracted according to Shamanin et al. [28]. The concentrations of free and bound phenolic compounds were determined using the Folin–Ciocalteu method, according to Özkan et al. [29]. Results were expressed as mg of gallic acid equivalents (GAE) per 100 g of dry weight (dw). The total phenolic content (TPC) was calculated as the sum of free and bound phenolics. Antioxidant activities were evaluated using ABTS, DPPH, and FRAP assays according to the methods of Re et al. [30], Singh et al. [31], and Benzie et al. [32], respectively. Results were expressed as mg TE/100 g dw.

### 2.5. In Vitro Starch Hydrolysis and Predicted Glycemic Index Determination

The in vitro predicted glycemic index (pGI) values of *Bandek* samples were assessed utilizing a glucose assay kit (Megazyme Int., Wicklow, Ireland), in accordance with the methodologies established by Goñi et al. [33] and Tekin et al. [34]. In summary, a 100 mg sample was weighed into 50 mL centrifuge tubes containing ten glass beads (5 mm in diameter). Following this, 2 mL of a 0.05 M hydrochloric acid (HCl) solution, supplemented with pepsin (5 mg/mL; Sigma, P7000, St. Louis, MO, USA), was added to each tube. The tubes were incubated at 37 °C in a shaking water bath for 30 min to simulate gastric digestion. After incubation, 4 mL of 0.5 M sodium acetate buffer (pH 5.2) and 1 mL of an enzyme solution containing pancreatin (0.104 g; Sigma, P7545, St. Louis, MO, USA) and amyloglucosidase (14.45 U; 3300 U/mL, Megazyme Int., Ireland) were added to each tube. The tubes were subsequently incubated horizontally at 37 °C in a shaking water bath to mimic intestinal digestion. Aliquots of 100 µL were collected at 0 and 90 min, transferred into Eppendorf tubes containing 1 mL of absolute ethanol, and centrifuged at 800× *g* for 10 min. The glucose concentration in the supernatant was quantified spectrophotometrically at 510 nm using glucose oxidase-peroxidase (GOPOD) reagent (Megazyme Int., Ireland). The hydrolysis index (HI) was calculated by comparing the results to those obtained from white bread, which served as the standard reference food (GI = 100). The predicted glycemic index was calculated using the equation proposed by Goñi et al. [33]:

(3) pGI=39.71+(0.549×HI)

### 2.6. Characterization of Volatile Organic Compounds (VOCs)

The extraction of volatile organic compounds was carried out using an optimized headspace solid-phase micro-extraction (HS-SPME) method. Before proceeding with the extraction, the samples (flour and the respective *Bandek*) were subjected to the cooking process according to the instructions provided by the partners from Morocco. In detail, a 50 g sample was combined with 50 g of distilled water and left to hydrate for 2–3 min. The mixture was then microwaved at 800 Watts for 1 min, stirred, and supplemented with 10–20 mL of water if required. A second microwave heating cycle (1 min, 800 Watts) was subsequently applied. The prepared product was then subjected to analytical evaluation. To generate the sample headspace, sample (1 g) was placed in 20 mL SPME glass vials (Chrom4 GmbH, Sulh, Germany) containing 1 gr of NaCl and 7 mL of a 20% (*w*/*v*) NaCl solution. *2-Octanol* (5 μL, 30 ppm, Sigma-Aldrich, St. Louis, MO, USA) was supplemented as an internal standard. To equilibrate the system, vials were incubated for 15 min at 60 °C to release free volatiles into the headspace. A SPME fiber, assembly 50/30 μm, divinylbenzene/carboxen/polydimethylsiloxane (Supelco, Bellefonte, PA, USA), was inserted into the vial’s headspace for 50 min at 60 °C for volatile extraction [35]. The fiber was then desorbed for 10 min at 250 °C within the inlet of the 8890 GC (Agilent, Santa Clara, CA, USA) equipped with an HP-INNOWAX capillary column (60 m × 0.25 mm, 0.25 μm film thickness), coupled to a 5977C MS detector (both Agilent, Technologies, Santa Clara, CA, USA). Helium was the carrier gas at a constant rate of 1 mL min^−1^. The analyses were performed with programmed temperatures: an initial temperature of 40 °C was maintained for 2 min, then raised from 40 to 200 °C at 4 °C/min, with the final temperature being maintained for 10 min. Mass spectrometric detection was performed in electron impact ionization (EI+) mode with an electron energy of 70 eV; the transfer line, ion source, and quadrupole temperatures were maintained at 280 °C, 290 °C and 150 °C, respectively, and mass spectra were acquired in full-scan mode over an *m*/*z* range of 40–300. The volatile organic compounds were identified by comparing the experimental mass spectra with those in the NIST/EPA/NIH Mass Spectral Database [36], using an MS match factor of at least 80%. The identification was also verified by comparing their linear retention indices (LRI), as determined in relation to the retention times of the C5–C29 n-alkane series, with the values reported in the literature [37,38]. The concentration of each volatile compound was calculated using the internal standard method [39,40]. The resulting semi-quantitative concentration data (in ng/g sample) for all identified VOCs were used for all subsequent statistical analyses. All analyses were performed in duplicate. During GC–MS analysis, a quality control (QC) sample was injected after every 10 samples to monitor instrument stability, repeatability, and analytical performance.

### 2.7. Statistical Analysis

Physicochemical, nutritional, and mineral analyses were conducted in triplicate, and results were expressed as mean ± standard deviation. Statistical analyses were performed using SPSS Statistics software (IBM SPSS Statistics, version 22.0, New York, NY, USA). Differences among samples were evaluated by one-way analysis of variance (ANOVA) followed by Tukey’s post hoc test. The differences in the color, β-glucan, protein, resistant starch, mineral content, phenolic content, and antioxidant capacities between the raw material and *Bandek* samples were evaluated by a paired *t*-test.

Characterization of Volatile Organic Compounds experiments were conducted in duplicate, and results were expressed as mean ± standard deviation. Differences among samples were evaluated by two-way ANOVA.

Prior to multivariate analysis, the VOC data were transformed and standardized to ensure comparability across compounds. Specifically, the data were first natural log-transformed using the log (1 + x) function and then standardized by z-score normalization (i.e., each variable was mean-centered and scaled to unit variance). The logarithmic transformation was applied to mitigate the large differences in the orders of magnitude among the abundances of the various volatile compounds, preventing the most concentrated compounds from dominating the analysis, while the addition of one prior to the logarithm allowed zero values to be handled. Both Principal Component Analysis (PCA) and hierarchical cluster analysis were then performed on the transformed and standardized VOC dataset using MATLAB (version 2024a, The MathWorks Inc., Natick, MA, USA). The hierarchical clustering and the associated heatmap were generated with the clustergram function (MATLAB Bioinformatics Toolbox v. 24.1), clustering both rows and columns using the Euclidean distance metric and the average linkage method.

## 3. Results and Discussion

### 3.1. Effect of Traditional Processing on Color Parameters and Browning Development in Bandek Samples

The color parameters of *Bandek* samples were significantly affected by the traditional processing steps, including steaming, drying, and roasting (Table 1). Processing into *Bandek* consistently reduced lightness (L*) and increased redness (a*), yellowness (b*), and the Browning Index (BI), reflecting the progressive development of browning reactions during thermal processing.

Among flour samples, Chifaa exhibited the highest L* value (88.70), reflecting the naturally lighter color of hull-less barley flour, whereas Faridur and Svevo showed the lowest values (80.19 and 80.20, respectively). Svevo HA displayed intermediate but significantly higher lightness (84.38) compared with the conventional durum wheat varieties. Regarding yellowness (b*), Faridur flour showed the highest value (24.86), while Chifaa exhibited the lowest (13.25), likely reflecting differences in pigment composition between these genotypes [41]. Browning Index values followed a similar trend, with Faridur flour presenting the highest BI (37.18) and Chifaa the lowest (16.35).

Following traditional processing, all *Bandek* samples exhibited reduced L* values and increased a*, b*, and BI values compared with their respective flours, highlighting the strong impact of thermal treatment on color development. Among *Bandek* samples, Svevo HA exhibited the highest L* value (75.40), suggesting a relatively lighter final product, whereas Faridur showed the lowest L* value (66.92), indicative of more pronounced browning, as confirmed by the highest BI value (56.06). The increase in redness was particularly evident in Faridur (a* = 6.65), while Svevo HA displayed the lowest a* value (4.34). Similarly, yellowness increased in all processed products, with Chifaa showing the highest b* value (28.46). The paired *t*-test was employed to evaluate the differences between the raw materials and corresponding *Bandek* samples produced from them. The results indicated significant differences between all of the color values of the raw materials and the corresponding *Bandek* products (*p* < 0.05).

These color modifications are mainly attributable to Maillard reactions and caramelization phenomena occurring during roasting, leading to the formation of brown pigments and flavor-active compounds [4,42]. The more pronounced browning observed in certain genotypes, particularly Faridur, may be related to differences in compositional and technological traits, including protein content, starch damage, particle size distribution, and the availability of reducing sugars, all of which can influence Maillard reactions during thermal processing. Faridur is characterized by softer kernel texture compared with Svevo, resulting in finer particles and modified starch properties that can affect color development during roasting [21]. Similar relationships between starch damage, thermal reactions, and crust browning have previously been reported in Svevo- and Faridur-based products [21].

### 3.2. The Characterization of Bandek Samples

The nutritional composition of *Bandek* samples revealed significant genotype-dependent variations and clear effects of processing (Table 2). A major distinguishing feature among samples was the β-glucan content, which was substantially higher in the barley-based *Bandek* (Chifaa: 6.03 g/100 g dw) compared to wheat-based samples (0.48–0.72 g/100 g dw), reflecting both the well-established role of barley as a major source of soluble dietary fibre [43,44] and the intrinsically high-fibre profile of the Chifaa cultivar. Processing resulted in modest, non-significant reductions in β-glucan content in Svevo (3.8%) and Faridur (5.9%), whereas significant decreases were observed in Svevo HA (11.1%) and Chifaa (14.8%) (*p* < 0.05). In Chifaa, β-glucan content decreased from 7.08 g/100 g dw in the raw flour to 6.03 g/100 g dw in *Bandek*. This reduction likely reflects the partial solubilization of the water-soluble β-glucan fraction during soaking and its subsequent removal with the soaking water and steam condensate, although the contribution of each processing step was not directly quantified. Nevertheless, Chifaa *Bandek* retained a substantial β-glucan concentration, consistent with the partial preservation of this component reported in thermally processed barley products [45].

Protein content significantly differed among the cereal genotypes (Table 2), with Chifaa showing the highest values in both flour and *Bandek* samples (18.01% and 14.27%). Svevo HA exhibited a slightly higher protein content than Svevo (16.10% vs. 15.04%), likely due to the trade-off between protein and starch accumulation, as this high-amylose genotype is characterized by a moderate reduction in starch content compared with the conventional Svevo cultivar [46]. Traditional *Bandek* processing resulted in an approximately 20% reduction in protein content in all genotypes, indicating that protein loss was mainly associated with the processing conditions rather than genotype-specific effects. This reduction may be related to soaking, which can promote the loss of soluble nitrogen compounds. Despite this decrease, measurable protein levels were retained in all *Bandek* samples, with values ranging from 11.46% to 14.27% under the conditions examined. Higher RS contents were observed in all *Bandek* samples compared with the corresponding raw flours. Raw flours showed very low RS values, close to the detection limit, with the exception of the high-amylose genotype Svevo HA, which exhibited markedly higher levels (6.66%). Following *Bandek* processing, all samples showed increased RS contents, reaching values of 1.72, 1.92 and 1.86% respectively in Svevo, Faridur, and Chifaa, while Svevo HA displayed a slight further increase up to 6.82%. The pronounced relative increase observed in conventional genotypes is likely associated with starch gelatinization during steaming followed by retrogradation occurring during cooling and drying, leading to the formation of enzyme-resistant starch structures [47]. In contrast, the limited increase observed in Svevo HA may be attributed to its already high RS content in the raw flour. Moreover, although RS in raw Svevo HA is predominantly present as RS2, processing into *Bandek* is expected to convert a substantial proportion of it into RS3 [48]. The t-test results indicated significant differences between the protein, β-glucan, and resistant starch contents of the raw materials and the corresponding *Bandek* samples (*p* < 0.05).

Iron and zinc contents were markedly higher in *Bandek* samples than in the corresponding flours. Iron concentration increased by approximately 44–110%, whereas zinc content showed a more moderate increase ranging from about 10% to 29%, depending on the genotype. Among the processed products, Svevo *Bandek* exhibited the highest iron (71.29 ppm) and zinc (41.19 ppm) contents, corresponding to increases of approximately 110% and 28%, respectively, compared with raw flour. Because no mineral-containing ingredients were added during processing, transfer from the traditional metal pan represents a plausible explanation for these increases. Previous studies have reported substantially higher Fe concentrations in foods cooked in iron utensils or steel woks and have demonstrated the migration of multiple elements, including Fe and Zn, from conventional and artisanal metal cookware [49,50,51,52]. In the present process, prolonged roasting (35–50 min), direct grain–pan contact, and continuous stirring may have promoted metal release through surface wear or abrasion, potentially contributing to the pronounced increase in Fe and, to a lesser extent, Zn. However, because the elemental composition and surface condition of the pan were not characterized and no migration controls were included, this hypothesis cannot be confirmed or quantified.

The results should be interpreted as compositional differences and not as evidence of mineral enrichment or increased mineral bioavailability. Future studies comparing traditional metal pans with inert cookware, together with elemental characterization of the utensils and mineral bioaccessibility analyses, are required to clarify the origin and nutritional relevance of these changes.

### 3.3. In Vitro Predicted Glycemic Index (pGI) and Hydrolysis Index (HI)

The in vitro starch digestibility analysis revealed significant differences among the *Bandek* samples (Table 2). Chifaa exhibited the lowest hydrolysis index (HI, 25.63) and estimated glycemic index (pGI, 53.78), followed by Svevo HA (HI, 38.29; pGI, 60.73), whereas Faridur showed the highest values (HI, 63.82; pGI, 74.75), indicating more rapid starch hydrolysis and potentially faster glucose release. Svevo displayed intermediate HI and pGI values of 52.58 and 68.58, respectively.

Based on the commonly used pGI thresholds, Chifaa would fall within the low-pGI category, Svevo HA and Svevo within the medium-pGI category, and Faridur within the high-pGI category [53]. However, because these values were estimated using an in vitro digestion assay, this classification should be interpreted as indicative of the potential glycemic response rather than as a direct assessment of the in vivo GI.

The comparison between Svevo HA and Chifaa highlights that resistant starch content and predicted glycemic response are not controlled by the same compositional factor. Chifaa contained less RS but retained a markedly higher β-glucan concentration than the wheat genotypes. The lower pGI of Chifaa is therefore likely attributable primarily to the viscosity-enhancing effect of β-glucans, which can limit enzyme diffusion and starch accessibility, while processing-induced resistant starch may provide an additional contribution [54,55]. By contrast, Svevo HA exhibited the highest RS level because of its intrinsically high-amylose starch, which is less susceptible to enzymatic hydrolysis and already contains a large resistant starch fraction before processing [56,57]. Thus, in Svevo HA the reduced starch digestibility appears to be driven mainly by starch composition and resistant starch, whereas in Chifaa soluble dietary fiber plays a more prominent role. This distinction also explains why the genotype with the highest resistant starch content did not necessarily exhibit the lowest pGI. The low pGI of Chifaa is likely primarily related to its high β-glucan content. β-Glucans can increase the viscosity of the food matrix and reduce enzyme accessibility to starch, thereby slowing starch hydrolysis and glucose release [54,55]. The RS formed during *Bandek* processing may have provided an additional contribution to its reduced starch digestibility. Similarly, the relatively low pGI of Svevo HA is consistent with its markedly higher RS content. High-amylose starches generally exhibit reduced susceptibility to enzymatic hydrolysis and are associated with attenuated postprandial glycemic responses [48,49]. This interpretation is further supported by the inverse relationship observed between RS content and pGI across the *Bandek* samples [58].

Nevertheless, the high estimated pGI of Faridur, despite its processing-induced increase in RS, indicates that RS content alone does not fully determine starch digestibility. Rather, the glycemic potential of *Bandek* appears to depend on the overall composition and structural properties of the cereal matrix, including starch characteristics and the presence of soluble dietary fiber.

These results demonstrate that the potential glycemic response of *Bandek* is strongly genotype-dependent and is modulated by the interaction between the composition of the raw material and traditional processing. In conventional genotypes, starch gelatinization during steaming, followed by retrogradation during cooling and drying, likely promoted the formation of RS3 and other less digestible starch structures. In Svevo HA, which already contained a high proportion of RS2 due to long chains of amylopectin in the raw flour, processing may have promoted partial conversion of native granular RS2 into retrograded RS3 [48]; however, the individual RS fractions were not directly quantified. Therefore, selecting genotypes with specific compositional traits, particularly high amylose or β-glucan contents, may represent an effective strategy for improving the nutritional and functional properties of traditional cereal-based foods such as *Bandek*.

### 3.4. Phenolic Content and Antioxidant Capacities

The phenolic profile of *Bandek* samples was significantly improved compared to the flours (Table 3). Total phenolic content (TPC) increased in all samples, reaching values between 484.30 and 501.72 mg GAE/100 g dw in *Bandek*. The highest values were observed in Chifaa and Faridur, indicating that both barley and certain wheat genotypes respond favorably to processing in terms of phenolic enrichment. The *t*-test results indicated that all of the *Bandek* samples had significantly higher free, bound, and total phenolic contents compared to those of the corresponding raw materials (*p* < 0.05).

A key observation is the increase in both free and bound phenolic fractions, with bound phenolics exceeding 250 mg GAE/100 g dw in all *Bandek* samples. This suggests that thermal processing promotes the release of phenolic compounds from the cereal matrix, likely through disruption of cell wall structures and cleavage of ester linkages [6]. Similar increases in phenolic extractability following thermal treatments have already been observed in barley [4]. These findings are particularly relevant from a nutritional standpoint, as phenolic compounds are associated with antioxidant, anti-inflammatory, and metabolic health benefits [59].

The enhancement of phenolic extractability was accompanied by a significant increase in antioxidant activity, as measured by ABTS, FRAP, and DPPH assays (Table 4). All *Bandek* samples showed higher antioxidant capacities than their corresponding flours, confirming the strong impact of processing. Among them, Chifaa exhibited the highest antioxidant activity across all assays (ABTS: 511.60; FRAP: 295.16; DPPH: 463.30 mg TE/100 g dw), reflecting its superior phenolic content. The *t*-test results indicated that all of the *Bandek* samples had significantly higher total antioxidant capacity values as determined by DPPH, ABTS, and FRAP assays compared to those of the corresponding raw materials (*p* < 0.05).

The extractability of both free and bound antioxidants increased after processing, with bound phenolics contributing substantially to the overall antioxidant capacity. These findings confirm the key role of bound phenolic compounds in determining the antioxidant potential of cereal-based foods, in agreement with previous studies on cereal antioxidants [5]. The enhanced antioxidant activity may be associated not only with the release of phenolic compounds from the cereal matrix during processing, but also with the formation of Maillard reaction products generated during roasting, which are known to exhibit antioxidant properties [60,61]. It should also be considered that the Folin–Ciocalteu assay is not specific for phenolics, and the observed increase in total phenolic content may partially reflect the formation of Maillard reaction products and other reducing compounds generated during roasting. Further studies based on chromatographic characterization of individual phenolic compounds, together with the evaluation of thermal contaminants such as acrylamide, would help to better clarify the chemical changes occurring during *Bandek* processing.

### 3.5. Volatile Organic Compounds

The volatile organic compounds (VOCs) identified and semi-quantified in wholemeal flours from Svevo, Svevo HA, Faridur, and the barley cultivar Chifaa, together with those detected in the corresponding *Bandek* products, are reported in Table S1. The effects of processing stage and cereal genotype were evaluated by two-way ANOVA (Table S2). A total of 64 VOCs were detected and classified as aldehydes (22 compounds), alcohols (7), terpenes and terpenoids (9), pyrazines (13), ketones (8), and other compounds (5). *Bandek* processing markedly altered the overall volatile profile, although the direction and magnitude of the changes differed among chemical classes. The total semi-quantitative VOC abundance increased from 874.06–1084.82 ng/g in the flours to 3378.38–7242.90 ng/g in the corresponding *Bandek* products, representing an approximately three- to eightfold increase, depending on genotype. This increase was driven mainly by aldehydes, pyrazines, and selected ketones and terpenoids, whereas several alcohols were less abundant after processing.

Pyrazines provided the clearest chemical signature associated with *Bandek* processing. Alkyl-substituted pyrazines were virtually absent from the raw flours, except for traces detected in Chifaa flour (13.4 ng/g), whereas their total abundance ranged from 270.21 to 448.67 ng/g in *Bandek*. Twelve or thirteen pyrazine derivatives were detected in the processed products, depending on the cereal genotype. These compounds included methyl-, dimethyl-, ethylmethyl-, and diethylmethyl-substituted pyrazines, which are commonly associated with the Maillard reaction and with roasted or toasted odor notes [61,62]. Their detection predominantly in *Bandek* is consistent with the thermal reactions occurring during roasting. Svevo- and Faridur-based *Bandek* showed the highest total pyrazine abundances (448.7 and 441.5 ng/g, respectively). Because roasting conditions were not instrumentally monitored, part of these differences may reflect variability in roasting intensity among samples; genotype-dependent differences in the availability of reducing sugars, amino compounds, or other reaction precursors may also have contributed.

Aldehydes were the most abundant VOC class in *Bandek*, ranging from 1151.60 to 5073.46 ng/g and accounting for approximately 34–70% of the total VOC abundance, compared with 113.49–138.85 ng/g in the corresponding flours. Fourteen aldehydes were detected in the *Bandek* samples, including heptanal, octanal, (E)-2-heptenal, (E)-2-octenal, 2-decenal, and furfural. Straight-chain and unsaturated aldehydes such as hexanal, heptanal, octanal, nonanal, (E)-2-octenal, (E,E)-2,4-nonadienal, and (E,E)-2,4-decadienal are commonly associated with lipid oxidation pathways. Hexanal, a major secondary oxidation product of linoleic acid, was considerably more abundant in *Bandek* than in the corresponding flours and reached its highest value in Faridur *Bandek* (576.5 ng/g) [63]. These patterns are consistent with increased lipid oxidation during processing; however, they do not establish differences among genotypes in lipoxygenase activity or fatty acid composition, which were not measured in this study.

Furfural was detected only in three processed products (Svevo HA, Chifaa, and Svevo) at concentrations ranging from 106.23 to 253.47 ng/g, whereas it was not detected in the raw flours or in Faridur *Bandek*. Furfural can arise from the thermal degradation of sugars and is commonly reported in thermally processed cereal products [64,65]. Its occurrence together with alkyl-substituted pyrazines is therefore consistent with non-enzymatic browning reactions during *Bandek* processing.

Ketones constituted the second most abundant class in *Bandek*, with total abundances ranging from 655.25 ng/g in Svevo to 1157.70 ng/g in Faridur. Nevertheless, the response of individual ketones was compound-specific. In particular, 2-octanone was more abundant in the raw flours (332.95–485.23 ng/g) than in the corresponding *Bandek* products (226.00–366.18 ng/g), suggesting partial loss through volatilization or further transformation during processing. Conversely, 3,5-octadien-2-one isomers were detected mainly, or at substantially higher abundances, in *Bandek*. These unsaturated ketones are consistent with secondary oxidation reactions involving unsaturated fatty acids [66].

Alcohols displayed an opposite overall trend. Their total abundance decreased from 212.52–467.00 ng/g in the raw flours to 115.48–411.41 ng/g in *Bandek*, mainly because 1-hexanol, 1-heptanol, and 1-pentanol were depleted or were no longer detected after processing. This reduction may reflect volatilization and/or further oxidation during roasting, although the relative contribution of these mechanisms was not directly assessed.

1-Octen-3-ol (mushroom-like, earthy) behaved in a matrix-dependent way on processing: present in Chifaa and Faridur flours (26.4 and 26.1 ng/g) and ~6-fold higher in the corresponding *Bandek* (149.8 and 152.1 ng/g), undetected in Svevo HA flour but the highest of all after processing (240.2 ng/g), and the reverse in Svevo (32.6 ng/g in flour, absent in the product). Since this C8 alcohol forms from linoleic acid via the genotype-dependent lipoxygenase/hydroperoxide-lyase pathway [67,68], its net increase in three of the four genotypes indicates formation during processing, mirroring the general rise in lipid-oxidation products in *Bandek*, whereas its loss in Svevo reflects volatilization and further transformation outweighing formation [69]. The final aroma is thus not a carry-over of the flour’s volatiles but the net outcome of matrix-dependent lipid oxidation and process-driven formation and loss.

The terpene and terpenoid fraction increased after processing, particularly in Svevo *Bandek*, which showed the highest total abundance in this class (885.67 ng/g). This profile was driven mainly by carvone, p-cymen-7-ol, and o-cymene, together with limonene, α-phellandrene, β-myrcene, and γ-terpinene. Their higher abundance in *Bandek* may reflect enhanced release from the cereal matrix or chemical transformation during processing. Although these compounds are commonly associated with herbaceous, citrus-like, or mint-like odor notes, their sensory contribution cannot be established without odor-activity or sensory analyses.

*Bandek* processing transformed the volatile profiles of the four cereal matrices. The processed products were characterized by the appearance of alkyl-substituted pyrazines and furfural, an increase in lipid-derived aldehydes and selected unsaturated ketones, and a decrease in several alcohols that were more abundant in the raw flours. The magnitude of these changes differed among genotypes: Svevo HA showed the highest total VOC and aldehyde abundances, whereas Svevo was distinguished by its greater terpene/terpenoid and pyrazine abundances. These results indicate that the composition of the starting cereal matrix modulates the volatile profile obtained after a common traditional processing procedure.

Taken together, the changes observed in resistant starch, phenolic compounds, antioxidant capacity, and volatile composition are likely to arise from different but sequentially connected stages of *Bandek* processing. Starch gelatinization during steaming, followed by molecular reassociation during drying and cooling, probably accounts for most of the increase in resistant starch through RS3 formation. In contrast, the increased extractability of phenolic compounds may result from the combined effects of hydrothermal treatment and subsequent heating, which can disrupt cell-wall structures and phenolic–matrix interactions. Roasting appears to play a particularly important role in the final chemical profile: in addition to promoting further release or transformation of phenolic compounds, it induces Maillard and other non-enzymatic browning reactions, as supported by the increased Browning Index and the appearance of alkyl-substituted pyrazines and furfural. Some Maillard-derived reducing compounds may also contribute to the higher Folin–Ciocalteu and antioxidant values measured in *Bandek*. Therefore, the increase in resistant starch and the changes in phenolic and volatile profiles should not be regarded as consequences of a single processing operation, but rather as the outcome of distinct and partially interacting transformations occurring throughout the steaming–drying/cooling–roasting sequence. Because intermediate products were not analyzed after each processing step; however, the relative contribution of the individual operations cannot be resolved experimentally in the present study.

### 3.6. Multivariate Analysis of Volatile Profiles

#### 3.6.1. Principal Component Analysis (PCA)

**Figure 1:**
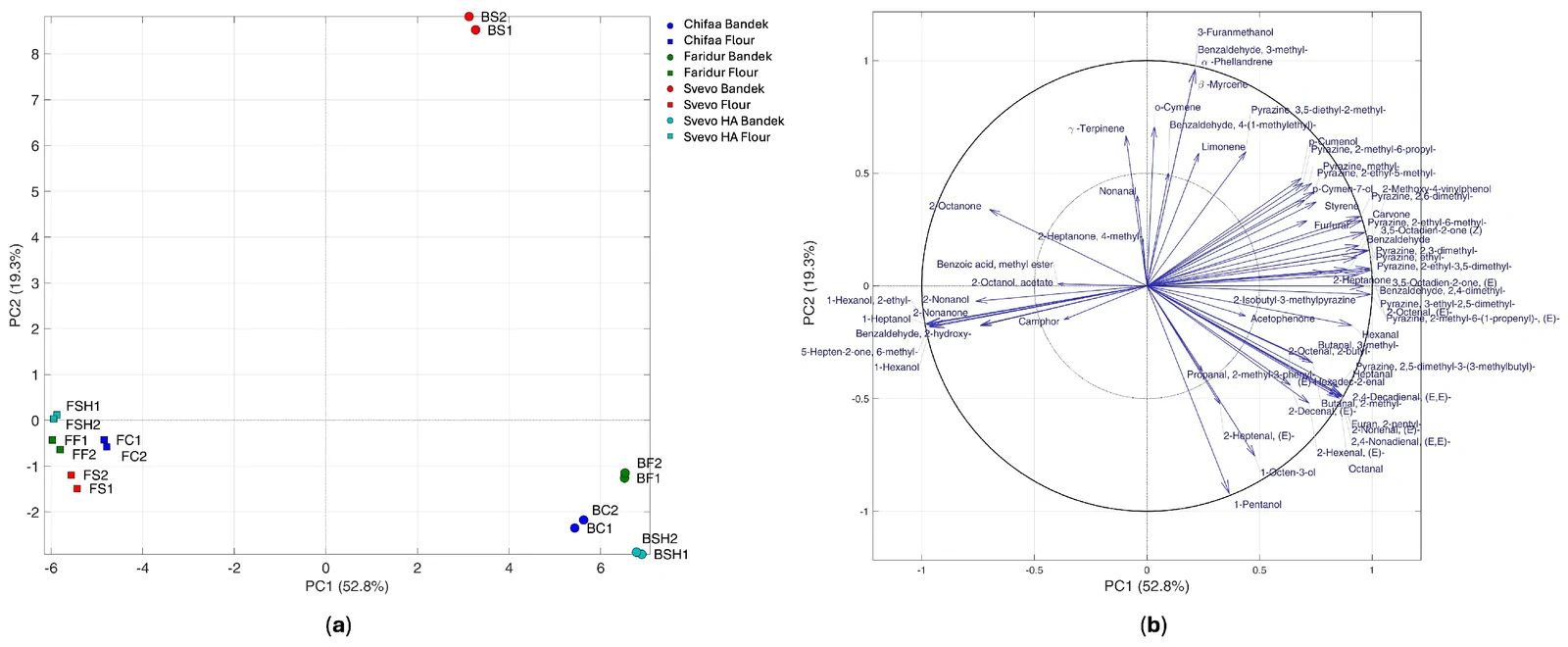
Principal component analysis (PCA) of the VOC data after log(1 + x) transformation and z-score standardization. (**a**) Scores of the samples along the first two principal components; (**b**) correlation circle plot of the VOC variables along the first two principal components. Sample codes consist of three parts: the first letter indicates the sample type (F = flour; B = *Bandek*), the following letter(s) indicate the cereal genotype (S = Svevo; SH = Svevo HA; F = Faridur; C = Chifaa), and the final digit (1–2) indicates the analytical replicate (e.g., FF1 = Faridur flour, replicate 1; BSH2 = Svevo HA *Bandek*, replicate 2).

Principal component analysis (PCA) was performed on the log1p-transformed and z-score-standardized VOC dataset to explore relationships among samples and variables and to assess analytical repeatability. The first two principal components explained 72.1% of the total variance, with PC1 and PC2 accounting for 52.8% and 19.3%, respectively (Figure 1). The score plot showed a clear separation between *Bandek* products and the corresponding raw flours along PC1: all *Bandek* samples were positioned on the positive side, whereas the flour samples clustered on the negative side. Additional differentiation among the *Bandek* samples was observed along PC2, particularly for Svevo *Bandek*, whose separation was associated with its higher terpene and terpenoid abundance. Svevo HA and Faridur *Bandek* were positioned on the positive side of PC1: Svevo HA was characterized by the highest total aldehyde abundance (5073.46 ng/g), whereas Faridur showed the highest total ketone abundance among the processed samples (1157.70 ng/g), driven mainly by the process-related 3,5-octadien-2-one isomers, together with the highest hexanal level (576.5 ng/g); Chifaa occupied an intermediate position among the processed samples. The loading plot indicated that positive PC1 loadings were mainly associated with pyrazines and thermally or oxidatively derived compounds, including furfural, (E)-2-octenal, hexanal, (E)-3,5-octadien-2-one, carvone, and p-cymen-7-ol. Conversely, 2-octanone, camphor, and 2-nonanol were associated with the negative side of PC1 and, therefore, with the flour samples. Positive PC2 loadings were primarily associated with o-cymene, γ-terpinene, limonene, and 4-(1-methylethyl)benzaldehyde, contributing to the separation of Svevo *Bandek*, whereas alcohols, short-chain unsaturated aldehydes, and branched-chain aldehydes were mainly associated with the negative PC2 direction. The analytical replicates of each matrix–treatment combination clustered closely, supporting the repeatability of VOC extraction and instrumental analysis. In conclusion, the PCA identified the distinction between raw flours and *Bandek* products as the main source of variation in the dataset, while also revealing secondary matrix-dependent differences among the processed products.

#### 3.6.2. Hierarchical Cluster Analysis

**Figure 2:**
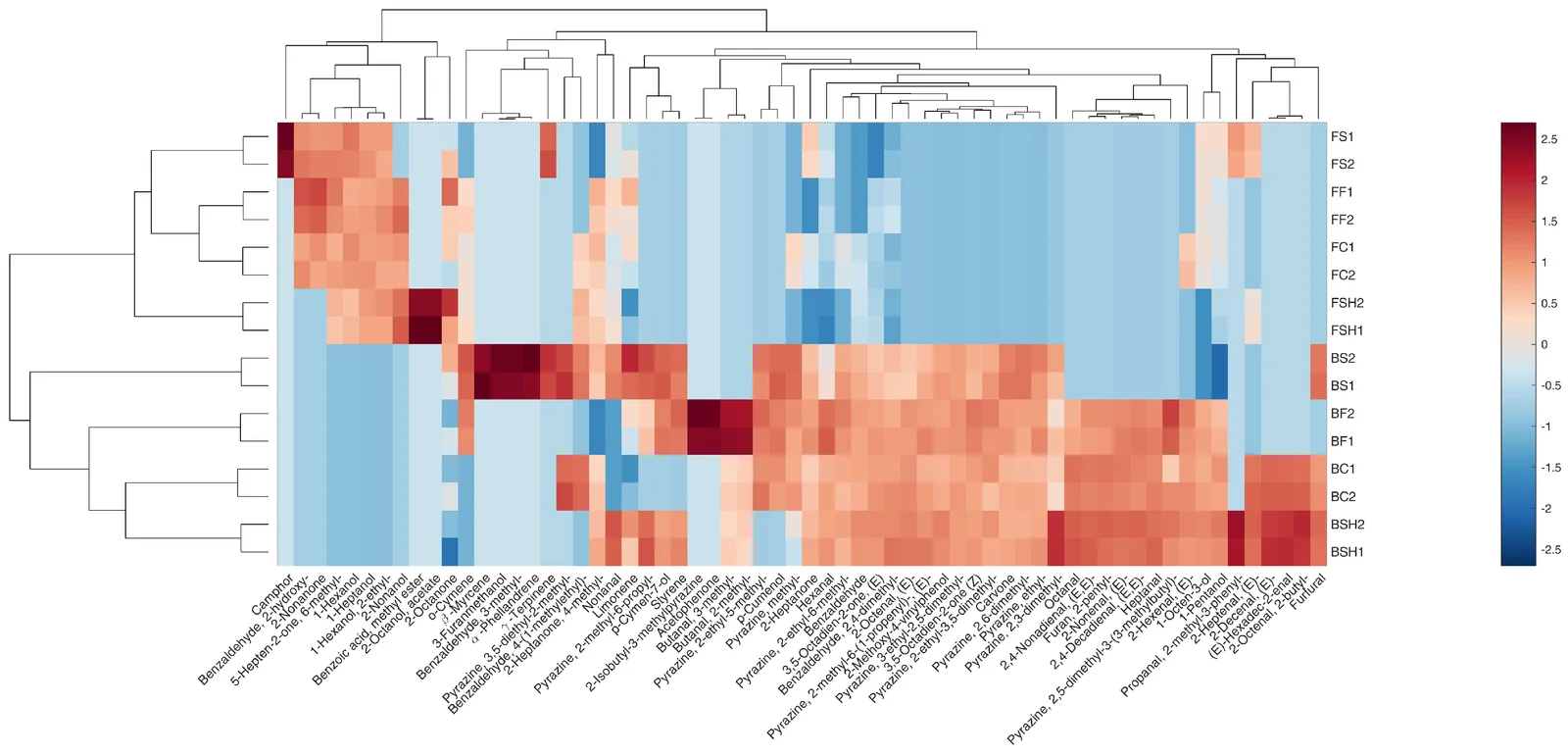
Cluster heatmap of the VOC data after log(1 + x) transformation and z-score standardization. Hierarchical clustering was performed using Euclidean distance and unweighted average linkage. Sample codes consist of three parts: the first letter indicates the sample type (F = flour; B = *Bandek*), the following letter(s) indicate the cereal genotype (S = Svevo; SH = Svevo HA; F = Faridur; C = Chifaa), and the final digit (1–2) indicates the analytical replicate (e.g., FF1 = Faridur flour, replicate 1; BSH2 = Svevo HA *Bandek*, replicate 2).

Consistent with the PCA, two-way hierarchical cluster analysis (HCA) of the transformed and standardized VOC data identified two main macro-clusters corresponding to the *Bandek* products and raw flours (Figure 2). Within the *Bandek* macro-cluster, Chifaa and Svevo HA formed the closest subgroup, characterized by higher standardized values for a cluster of compounds associated with the processed matrices; Faridur joined this subgroup at a greater linkage distance, whereas Svevo was the most distinct among the processed samples, consistent with its higher standardized values for several terpenes and terpenoids and with its separation along PC2 in the PCA. The flour macro-cluster showed a predominantly low standardized abundance of compounds associated with thermal processing and was further divided into matrix-specific subgroups. The compound dendrogram identified three broad abundance patterns: a cluster associated primarily with *Bandek*, comprising pyrazines, furfural, lipid-derived aldehydes, and selected unsaturated ketones; an intermediate cluster dominated by monoterpenes and terpenoids, with higher values in Faridur and Svevo *Bandek*; and a cluster containing compounds more closely associated with the background VOC profiles of the raw flours, including several alcohols and camphor. The close clustering of analytical replicates further confirmed analytical consistency. Overall, the HCA supported the primary separation between flours and *Bandek* products observed in the PCA and confirmed the presence of secondary matrix-dependent differences, particularly in the terpene and terpenoid profiles of the processed products.

## 4. Conclusions

This study shows that traditional *Bandek* processing substantially modifies the nutritional, functional, and physicochemical properties of barley and durum wheat, although the magnitude of the response depends on the cereal genotype. Under the conditions examined, processing increased extractable phenolic compounds and antioxidant capacity, promoted resistant starch formation, and lowered the estimated in vitro GI. Chifaa barley retained a high β-glucan content and showed the lowest pGI, whereas high-amylose Svevo produced the highest resistant starch content among the wheat-based products. These findings indicate that genotype selection and traditional processing act jointly in determining the nutritional characteristics of *Bandek*.

Processing also markedly reshaped the volatile profile, increasing total volatile organic compounds three- to eight-fold and promoting the formation of pyrazines, furfural, aldehydes, and other compounds associated with Maillard reactions, caramelization, and lipid oxidation. The genotype-dependent volatile patterns provide a chemical basis for differentiating the aroma profiles of the four *Bandek* products. However, the contribution of individual compounds to perceived aroma and consumer acceptance should be confirmed through sensory evaluation.

From a product-development perspective, the results indicate that different cereal matrices may be selected according to the desired nutritional or quality target rather than identifying a single optimal genotype for *Bandek* production. Chifaa barley appears particularly promising for β-glucan-rich *Bandek* products with reduced starch digestibility, as it retained a high β-glucan content (6.03%) after processing and exhibited the lowest pGI (53.78). Conversely, Svevo HA represents the most promising material when resistant starch enrichment is the primary objective, combining the highest RS content (6.82%) with a relatively low pGI (60.73). Svevo and Faridur *Bandek* showed the highest abundances of roasting-associated pyrazines, suggesting their potential interest for products in which the characteristic toasted volatile profile of traditional *Bandek* is particularly relevant. However, this latter interpretation requires confirmation through sensory and odor-activity analyses. Overall, these genotype-specific responses indicate that cereal selection can be tailored to emphasize different nutritional or aroma-related characteristics while preserving the traditional *Bandek* processing approach.

Further work should examine nutrient bioaccessibility and sensory quality and validate the physiological effects in human studies.

To conclude, combining nutritionally targeted cereal germplasm with culturally rooted *Bandek* processing offers a promising route to cereal foods with improved functional characteristics and distinctive volatile profiles. This approach may support the scientific valorization of Moroccan food heritage and the development of locally relevant cereal products, provided that nutritional benefits, sensory acceptance, and process reproducibility are confirmed in subsequent studies.

## Figures and Tables

**Table 1:** Color values of *Bandek* samples.

		L*	a*	b*	BI
Flour	Svevo	80.20 ± 0.43 ^cX^	1.81 ± 0.12 ^aY^	22.94 ± 0.10 ^bY^	34.20 ^b^
	Faridur	80.19 ± 0.58 ^cX^	1.51 ± 0.18 ^aY^	24.86 ± 0.55 ^aY^	37.18 ^a^
	Svevo HA	84.38 ± 0.44 ^bX^	1.02 ± 0.16 ^bY^	19.75 ± 0.18 ^cY^	26.64 ^c^
	Chifaa	88.70 ± 0.20 ^aX^	0.88 ± 0.04 ^bY^	13.25 ± 0.18 ^dY^	16.35 ^d^
*Bandek* **	Svevo	72.48 ± 0.10 ^BY^	4.76 ± 0.03 ^CX^	27.54 ± 0.14 ^BX^	50.95 ^B^
	Faridur	66.92 ± 0.27 ^CY^	6.65 ± 0.12 ^AX^	26.53 ± 0.31 ^BX^	56.06 ^A^
	Svevo HA	75.40 ± 0.22 ^AY^	4.34 ± 0.13 ^DX^	24.27 ± 0.57 ^CX^	41.80 ^C^
	Chifaa	72.72 ± 0.19 ^BY^	5.16 ± 0.03 ^BX^	28.46 ± 0.15 ^AX^	53.11 ^AB^

L*: Lightness—ranges from 0 (black) to 100 (white); indicates the brightness of the sample. a*: Red/Green Index—positive values indicate red tones; negative values indicate green tones. b*: Yellow/Blue Index—positive values indicate yellow tones; negative values indicate blue tones. BI: Browning Index; higher values indicate darker (more browned) samples. Data are expressed as mean ± standard deviation. Mean values within each column followed by different letters are significantly different (*p* < 0.05). Flour samples (wheat and barley; a–d) and *Bandek* samples (A–D) were evaluated separately. Means with different letters (X and Y) are significantly different (*p* < 0.05) between the raw material and *Bandek* samples. ** Prepared from each wheat and barley variety.

**Table 2:** The nutritional analysis of *Bandek* samples.

Samples	β-Glucan	Protein Content (%)	Resistant Starch (%)	Fe (ppm)	Zn (ppm)	HI	pGI
	(g/100 g dw)						
Flour							
Svevo	0.52 ± 0.01 ^cY^	15.04 ± 0.44 ^bcX^	0.34 ± 0.02 ^cY^	33.90 ± 0.43 ^aY^	32.06 ± 0.62 ^aY^		
Faridur	0.51 ± 0.01 ^cY^	14.52 ± 0.42 ^cX^	0.52 ± 0.01 ^bY^	33.41 ± 0.57 ^aY^	31.46 ± 0.65 ^abY^		
Svevo HA	0.81 ± 0.01 ^bX^	16.10 ± 0.23 ^bX^	6.66 ± 0.09 ^aY^	32.19 ± 0.44 ^abY^	28.10 ± 0.14 ^cY^		
Chifaa	7.08 ± 0.11 ^aX^	18.01 ± 0.71 ^aX^	0.42 ± 0.01 ^bcY^	31.35 ± 0.49 ^bY^	29.84 ± 0.48 ^bcY^		
*Bandek* *							
Svevo	0.50 ± 0.02 ^CY^	12.03 ± 0.13 ^BCY^	1.72 ± 0.02 ^DX^	71.29 ± 0.41 ^AX^	41.19 ± 0.27 ^AX^	52.58 ± 0.91 ^B^	68.58 ± 0.50 ^B^
Faridur	0.48 ± 0.02 ^CY^	11.46 ± 0.53 ^CY^	1.92 ± 0.02 ^BX^	56.02 ± 0.17 ^BX^	36.30 ± 0.42 ^BX^	63.82 ± 0.41 ^A^	74.75 ± 0.23 ^A^
Svevo HA	0.72 ± 0.02 ^BY^	12.80 ± 0.38 ^BY^	6.82 ± 0.01 ^AX^	57.16 ± 0.23 ^BX^	31.23 ± 0.33 ^CX^	38.29 ± 0.45 ^C^	60.73 ± 0.24 ^C^
Chifaa	6.03 ± 0.02 ^AY^	14.27 ± 0.35 ^AY^	1.86 ± 0.02 ^CX^	45.20 ± 0.28 ^CX^	32.67 ± 0.47 ^CX^	25.63 ± 0.24 ^D^	53.78 ± 0.13 ^D^

Data are expressed as mean ± standard deviation; mean values in each column with different letters are significantly different (*p* < 0.05). Flour samples (wheat and barley a–c) and *bandek* samples (A–D) were evaluated separately. Means with different letters (X and Y) are significantly different (*p* < 0.05) between the raw material and *Bandek* samples. * Prepared from each wheat and barley variety. HI: hydrolysis index; pGI: in vitro predicted glycemic index.

**Table 3:** Phenolic contents of *Bandek* samples.

		Free	Bound	Total **
Flour	Svevo	218.30 ± 1.55 ^cS^	221.99 ± 2.16 ^fS^	440.29 ± 0.71 ^zS^
	Faridur	224.13 ± 0.96 ^bS^	225.18 ± 2.85 ^efS^	449.30 ± 3.47 ^yS^
	Svevo HA	221.67 ± 1.05 ^bS^	223.30 ± 3.07 ^fS^	444.97 ± 3.12 ^yzS^
	Chifaa	244.70 ± 0.98 ^aS^	230.02 ± 1.33 ^eS^	474.71 ± 0.37 ^xS^
*Bandek* *	Svevo	230.74 ± 0.88 ^BR^	254.78 ± 1.06 ^ER^	485.52 ± 0.98 ^YR^
	Faridur	243.52 ± 1.23 ^AR^	254.08 ± 1.78 ^ER^	497.60 ± 0.60 ^XR^
	Svevo HA	231.18 ± 1.82 ^BR^	253.13 ± 1.80 ^ER^	484.30 ± 2.26 ^YR^
	Chifaa	246.47 ± 1.87 ^AR^	255.25 ± 2.02 ^ER^	501.72 ± 3.51 ^XR^

Data are expressed as mean ± SD of triplicate measurements. Flour and *Bandek* samples were analyzed separately. Different letters within the same column indicate significant differences among flour samples for the same phenolic fraction (free, a–c; bound, e and f; total, x–z) and among *Bandek* samples for the corresponding fraction (free, A–B; bound, E; total, X and Y), according to Tukey’s post hoc test (*p* < 0.05). Means with different letters (R and S) are significantly different (*p* < 0.05) between the raw material and *Bandek* samples. * Prepared from each wheat and barley variety. ** Total phenolic content was calculated as the sum of free and bound fractions and expressed as mg gallic acid equivalents (GAE)/100 g dry weight (dw). It should be emphasized that the present study was designed to compare raw cereal matrices with their corresponding *Bandek* products and did not include cereals processed by alternative traditional or modern technologies. Therefore, the observed compositional changes should be interpreted specifically within the *Bandek* processing conditions examined here and should not be considered evidence of nutritional superiority over other cereal-processing methods.

**Table 4:** Antioxidant capacities (ABTS, FRAP, and DPPH) of *Bandek* samples.

		Samples	ABTS	FRAP	DPPH
Flour	Free	Svevo	99.47 ± 0.65 ^c^	40.86 ± 0.35 ^c^	98.54 ± 1.11 ^c^
		Faridur	105.08 ± 0.51 ^b^	47.49 ± 1.20 ^b^	103.86 ± 1.14 ^b^
		Svevo HA	99.51 ± 0.99 ^c^	41.55 ± 0.96 ^c^	100.52 ± 1.49 ^bc^
		Chifaa	210.74 ± 0.34 ^a^	135.76 ± 0.61 ^a^	220.71 ± 1.50 ^a^
	Bound	Svevo	164.09 ± 1.00 ^f^	95.39 ± 0.47 ^h^	129.30 ± 1.66 ^h^
		Faridur	158.40 ± 1.02 ^g^	100.05 ± 1.33 ^g^	191.48 ± 1.70 ^f^
		Svevo HA	165.86 ± 0.99 ^f^	104.64 ± 0.53 ^f^	175.90 ± 1.08 ^g^
		Chifaa	233.02 ± 2.03 ^e^	141.73 ± 0.74 ^e^	226.13 ± 2.26 ^e^
	Total **	Svevo	263.56 ± 1.51 ^yS^	136.25 ± 0.42 ^zS^	227.85 ± 1.47 ^tS^
		Faridur	263.48 ± 1.35 ^yS^	147.54 ± 2.35 ^yS^	295.34 ± 2.69 ^yS^
		Svevo HA	265.38 ± 1.97 ^yS^	146.19 ± 1.43 ^yS^	276.42 ± 2.55 ^zS^
		Chifaa	443.76 ± 1.69 ^xS^	277.49 ± 0.14 ^xS^	446.84 ± 1.99 ^xS^
*Bandek* *	Free	Svevo	138.60 ± 0.47 ^C^	95.86 ± 0.55 ^D^	149.13 ± 1.04 ^D^
		Faridur	205.15 ± 1.56 ^B^	138.29 ± 0.34 ^B^	207.25 ± 1.06 ^B^
		Svevo HA	139.06 ± 1.14 ^C^	126.16 ± 1.35 ^C^	185.98 ± 1.61 ^C^
		Chifaa	228.64 ± 3.21 ^A^	146.18 ± 1.11 ^A^	221.69 ± 2.06 ^A^
	Bound	Svevo	254.19 ± 0.93 ^G^	122.92 ± 0.87 ^H^	156.59 ± 1.04 ^H^
		Faridur	267.04 ± 1.95 ^F^	143.50 ± 0.89 ^F^	209.58 ± 1.80 ^F^
		Svevo HA	271.62 ± 2.38 ^F^	129.77 ± 0.45 ^G^	188.79 ± 1.05 ^G^
		Chifaa	282.97 ± 3.11 ^E^	148.98 ± 0.44 ^E^	241.61 ± 2.10 ^E^
	Total **	Svevo	392.79 ± 1.38 ^TR^	218.78 ± 1.20 ^TR^	305.72 ± 1.04 ^TR^
		Faridur	472.20 ± 3.26 ^YR^	281.78 ± 0.56 ^YR^	416.83 ± 2.26 ^YR^
		Svevo HA	410.69 ± 3.47 ^ZR^	255.94 ± 0.90 ^ZR^	374.77 ± 2.43 ^ZR^
		Chifaa	511.60 ± 0.53 ^XR^	295.16 ± 1.10 ^XR^	463.30 ± 3.78 ^XR^

Data are expressed as mean ± SD of triplicate measurements. Flour and *Bandek* samples were analyzed separately. Different letters within the same column indicate significant differences among flour samples for the same fraction (free, a–c; bound, e–h; total, t, x–z) and among *Bandek* samples for the same fraction (free, A–D; bound, E–H; total, T, X–Z), according to Tukey’s post hoc test (*p* < 0.05). Means with different letters (R and S) are significantly different (*p* < 0.05) between the raw material and *Bandek* samples. * Prepared from each wheat and barley variety. ABTS: 2,2′-azino-bis (3-ethylbenzothiazoline-6-sulfonic acid); DPPH: 2,2-diphenyl-1-picrylhydrazyl radical scavenging activity; FRAP: ferric reducing antioxidant power. ** Total antioxidant capacity calculated as the sum of free and bound fractions, expressed as mg TE/100 g dw.

## Data Availability

The original contributions presented in this study are included in the article/Supplementary Material. Further inquiries can be directed to the corresponding authors.

## Supplementary Materials

The following supporting information can be downloaded at: https://www.mdpi.com/article/10.3390/foods15183250/s1, Table S1: Volatile Organic Compounds identified in flours (F) and *Bandek* (B); Table S2: Two-way ANOVA applied to volatolomic data.
