# Supplementary material for: Effects of Traditional Moroccan Bandek Processing on Nutritional Composition, In Vitro Starch Digestibility, and Volatile Profiles of Barley and Durum Wheat Genotypes
Source: Foods. 2026 Sep 14;15(18):3250. doi: 10.3390/foods15183250 (PMC13606764; doi:10.3390/foods15183250)
Supplement: Supplementary file 1 [file foods-15-03250-s001.zip › foods-4502609-supplementary.pdf]

Table S1. Volatile Organic Compounds identified in flours (F) and *Bandek* (B).

Volatile Organic Compounds (ng/g)	F- SVEVO		F-FARIDUR		F- SVEVO HA		F- CHIFAA		B- SVEVO		B- FARIDUR		B- SVEVO HA		B- CHIFAA	
		±sd		*±sd*		*±sd*		*±sd*		*±sd*		*±sd*		*±sd*		*±sd*
**ALDEHYDES**																
(E)-Hexadec-2-enal	nd	nd	nd	nd	nd	nd	nd	nd	nd	nd	nd	nd	195.46	33.17	73.49	12.47
2,4-Decadienal, (E,E)-	nd	nd	nd	nd	nd	nd	nd	nd	nd	nd	128.83	21.86	199.19	33.8	104.22	17.69
2,4-Nonadienal, (E,E)-	nd	nd	nd	nd	nd	nd	nd	nd	nd	nd	124.91	21.2	396.71	67.32	213.24	36.19
2-Decenal, (E)-	nd	nd	nd	nd	nd	nd	nd	nd	nd	nd	0	0	279.73	47.47	110.51	18.75
2-Heptenal, (E)-	26.4	4.85	nd	nd	7.59	1.39	nd	nd	nd	nd	0	0	149.05	25.3	137.02	23.25
2-Hexenal, (E)-	nd	nd	nd	nd	nd	nd	8.55	1.57	nd	nd	21.69	3.68	20.76	3.52	18.45	3.13
2-Nonenal, (E)-	nd	nd	nd	nd	nd	nd	nd	nd	nd	nd	110.59	18.77	181.49	30.8	122.23	20.74
2-Octenal, (E)-	nd	nd	nd	nd	nd	nd	nd	nd	59.96	10.18	278.91	47.33	554.86	94.16	378.39	64.21
2-Octenal, 2-butyl-	nd	nd	nd	nd	nd	nd	nd	nd	nd	nd	nd	nd	234.93	39.87	77.86	13.21
Benzaldehyde	nd	nd	nd	nd	11.82	2.17	12.81	2.35	nd	nd	nd	nd	587.2	99.65	323.81	54.95
Benzaldehyde, 2,4-dimethyl-	17.53	3.22	43.62	8.02	14.23	2.62	20	3.68	168	28.51	270.15	45.85	416.45	70.67	312.86	53.09
Benzaldehyde, 2-hydroxy-	8.59	1.58	12.84	2.36	nd	nd	6.87	1.26	nd	nd	nd	nd	nd	nd	nd	nd
Benzaldehyde, 3-methyl-	nd	nd	nd	nd	nd	nd	nd	nd	107.52	18.25	nd	nd	nd	nd	nd	nd
Benzaldehyde, 4-(1-methylethyl)-	nd	nd	nd	nd	51.52	9.47	23.34	4.29	227.15	38.55	nd	nd	nd	nd	308.27	52.32
Butanal, 2-methyl-	nd	nd	nd	nd	nd	nd	nd	nd	nd	nd	76.92	13.05	3.72	0.63	4.48	0.76
Butanal, 3-methyl-	nd	nd	nd	nd	nd	nd	nd	nd	nd	nd	59.32	10.07	3.13	0.53	3.15	0.54
Furfural	nd	nd	nd	nd	nd	nd	nd	nd	213.91	36.3	nd	nd	253.47	43.02	106.23	18.03
Heptanal	nd	nd	nd	nd	nd	nd	nd	nd	0	0	58.58	9.94	96.32	16.35	61.41	10.42
Hexanal	50.09	9.21	33.61	6.18	7.62	1.4	30.06	5.53	91.1	15.46	576.45	97.83	296.07	50.24	258.22	43.82
Nonanal	14.15	2.6	32.83	6.04	22.37	4.11	11.85	2.18	283.96	48.19	nd	nd	628.14	106.6	nd	nd
Octanal	nd	nd	nd	nd	nd	nd	nd	nd	nd	nd	42.04	7.13	222.54	37.77	122.45	20.78
Propanal, 2-methyl-3-phenyl-	22.08	4.06	nd	nd	nd	nd	nd	nd	nd	nd	nd	nd	354.23	60.12	nd	nd
**Total**	**138.85**		**122.9**		**115.14**		**113.49**		**1151.6**		**1748.41**		**5073.46**		**2736.28**	
**ALCOHOLS**																

1-Heptanol	130.57	24	110.59	20.33	109.71	20.17	126.48	23.25	nd	nd	nd	nd	nd	nd	nd	nd
1-Hexanol	252.51	46.42	100.54	18.48	56.15	10.32	165.5	30.43	nd	nd	nd	nd	nd	nd	nd	nd
1-Hexanol, 2-ethyl-	18.28	3.36	23.13	4.25	20.58	3.78	18.3	3.37	nd	nd	nd	nd	nd	nd	nd	nd
1-Octen-3-ol	32.57	5.99	26.11	4.8	nd		26.35	4.84	nd	nd	152.13	25.82	240.17	40.76	149.76	25.42
1-Pentanol	33.07	6.08	19.05	3.5	8.78	1.61	18.35	3.37	nd	nd	76.71	13.02	171.24	29.06	118.46	20.1
2-Nonanol	nd	nd	14.68	2.7	17.3	3.18	8.52	1.57	nd	nd	nd	nd	nd	nd	nd	nd
3-Furanmethanol	nd	nd	nd	nd	nd	nd	nd	nd	115.48	19.6	nd	nd	nd	nd	nd	nd
**Total**	**467**		**294.1**		**212.52**		**363.5**		**115.48**		**228.84**		**411.41**		**268.22**	
**TERPENES**																
α-Phellandrene	nd		nd		nd		nd		8.34	1.42	nd		nd		nd	
β-Myrcene	nd		nd		nd		nd		8.25	1.4	nd		nd		nd	
γ-Terpinene	11.44	2.1	nd		nd		nd		15.52	2.63	nd		nd		nd	
Camphor	35.31	6.49	nd		nd		nd		nd		nd		nd		nd	
Carvone									396.02	67.21	208.72	35.42	127.13	21.58	105.64	17.93
Limonene	40.79	7.5	54.51	10.02	26.85	4.94	43.71	8.04	93.15	15.81	44.34	7.53	63.66	10.8	26.5	4.5
o-Cymene			11	2.02	8.44	1.55	6.92	1.27	90.19	15.31	51.31	8.71			0	0
p-Cumenol									81.82	13.89	55.77	9.46			34.08	5.78
p-Cymen-7-ol									192.37	32.65	109.56	18.59	55.5	9.42		
**Total**	**87.54**		**65.51**		**35.28**		**50.63**		**885.67**		**469.71**		**246.29**		**166.22**	
**PYRAZINE**																
Pyrazine, 2,3-dimethyl-	nd	nd	nd	nd	nd	nd	nd	nd	13.67	4.56	7.24	4.16	89.64	2.15	6.06	1.95
Pyrazine, 2,5-dimethyl-3-(3-methylbutyl)-	nd	nd	nd	nd	nd	nd	nd	nd	0	0	26.12	6.1	14.36	3.44	8.33	5.62
Pyrazine, 2,6-dimethyl-	nd	nd	nd	nd	nd	nd	nd	nd	115.76	3.78	48.39	8.92	47.11	11.19	37.56	9.52
Pyrazine, 2-ethyl-3,5-dimethyl-	nd	nd	nd	nd	nd	nd	nd	nd	18.47	4.44	26.37	6.46	18.91	4.54	17.74	9.57
Pyrazine, 2-ethyl-5-methyl-	nd	nd	nd	nd	nd	nd	nd	nd	34.81	8.37	43.82	10.8	0	0	34.64	9.73
Pyrazine, 2-ethyl-6-methyl-	nd	nd	nd	nd	nd	nd	4.42	0.81	34.11	8.19	48.41	11.6	28.67	6.89	25.32	6.12
Pyrazine, 2-methyl-6-(1-propenyl)-, (E)-	nd	nd	nd	nd	nd	nd	nd	nd	12.2	2.93	21.61	5.11	25.08	6.01	15.28	3.2
Pyrazine, 2-methyl-6-propyl-	nd	nd	nd	nd	nd	nd	nd	nd	41.49	9.96	6.15	1.47	35.7	8.58	nd	nd
Pyrazine, 3,5-diethyl-2-methyl-	nd	nd	nd	nd	nd	nd	nd	nd	31.92	7.44	0	0	nd	nd	21.53	6.63

Pyrazine, 3-ethyl-2,5-dimethyl-	nd	nd	nd	nd	nd	nd	nd	nd	64.32	1.54	81.64	2.14	71.21	1.77	63.75	14.92
Pyrazine, ethyl-	nd	nd	nd	nd	nd	nd	nd	nd	19.42	4.68	17.34	3.9	15.12	3.63	10.61	2.02
Pyrazine, methyl-	nd	nd	nd	nd	nd	nd	8.99	1.65	62.5	1.65	31.91	9.08	4.07	2.55	29.39	7.98
2-Isobutyl-3-methylpyrazine	nd	nd	nd	nd	nd	nd	nd	nd	nd	nd	82.56	19.81	nd	nd	nd	nd
**Total**	**0**		**0**		**0**		**13.41**		**448.67**		**441.55**		**349.88**		**270.21**	
**KETONES**																
2-Heptanone	21.96	4.04	nd	nd	nd	nd	7.2	1.32	35.14	5.96	59.81	10.15	39.85	6.76	26.35	4.47
2-Heptanone, 4-methyl-	nd	nd	9.13	1.68	6.77	1.25	8.52	1.57	8.7	1.48	nd	nd	10.47	1.78	7.44	1.26
2-Nonanone	7.79	1.43	14.47	2.66	nd	nd	6.46	1.19	nd	nd	nd	nd	nd	nd	nd	nd
2-Octanone	344.21	63.28	429.05	78.88	485.23	89.21	332.95	61.21	366.18	62.14	279.78	47.48	226	38.35	286.63	48.64
3,5-Octadien-2-one (Z)	nd	nd	nd	nd	nd	nd	nd	nd	86.56	14.69	346.65	58.83	174.67	29.64	145.99	24.78
3,5-Octadien-2-one, (E)	nd	nd	11.56	2.13	10.77	1.98	7.82	1.44	158.69	26.93	401.04	68.06	498.93	84.67	408	69.24
5-Hepten-2-one, 6-methyl-	17.49	3.22	19.26	3.54	8.34	1.53	12.07	2.22	nd	nd	nd	nd	nd	nd	nd	nd
Acetophenone	nd	nd	nd	nd	nd	nd	nd	nd	nd	nd	70.43	11.95	nd	nd	nd	nd
**Total**	**391.44**		**483.47**		**511.12**		**375.02**		**655.25**		**1157.7**		**949.92**		**874.41**	
**OTHERS**																
2-Methoxy-4-vinylphenol	nd	nd	nd	nd	nd	nd	nd	nd	106.85	18.13	111.59	18.94	175.76	29.83	70.94	12.04
Styrene	nd	nd	nd	nd	nd	nd	nd	nd	14.86	2.52	16.28	2.76	11.07	1.88	nd	
2-Pentyl furan									nd		78.37	13.3	196.1	33.28	131.69	22.35
**Total**									**121.71**		**206.24**		**328.93**		**202.63**	
**ESTERS**																
2-Octanol acetate	nd	nd	nd	nd	nd	nd	nd	nd	nd	nd	nd	nd	12.6	1.64	nd	nd
Benzoic acid methyl ester	nd	nd	nd	nd	nd	nd	nd	nd	nd	nd	nd	nd	12.51	1.63	nd	nd
**Total**													**25.11**			

nd: not detected; sd: standard deviation.

Table S2. Two-way ANOVA applied on volatolomic data.

	Two Way ANOVA	
	**Flours**	**Product**
**ALDEHYDES**		
(E)-Hexadec-2-enal	*	**
2,4-Decadienal, (E,E)-	*	***
2,4-Nonadienal, (E,E)-	*	***
2-Decenal, (E)-	*	**
2-Heptenal, (E)-		
2-Hexenal, (E)-	**	**
2-Nonenal, (E)-	*	***
2-Octenal, (E)-		****
2-Octenal, 2-butyl-	*	**
Benzaldehyde	*	****
Benzaldehyde, 2,4-dimethyl-		****
Benzaldehyde, 2-hydroxy-	*	***
Benzaldehyde, 3-methyl-	*	
Benzaldehyde, 4-(1-methylethyl)-		
Butanal, 2-methyl-	*	**
Butanal, 3-methyl-	*	**
Furfural	*	***
Heptanal	*	***
Hexanal		****
Nonanal		
Octanal	*	***
Propanal, 2-methyl-3-phenyl-		
**ALCOHOLS**		
1-Heptanol		****
1-Hexanol		****

	Two Way ANOVA	
	Flours	Product
1-Hexanol, 2-ethyl-		****
1-Octen-3-ol		
1-Pentanol		
2-Nonanol	*	***
3-Furanmethanol	*	
**TERPENES**		
α-Phellandrene	*	
β-Myrcene	*	
γ-Terpinene	****	
Camphor	*	
Carvone		****
Limonene		
o-Cymene		
p-Cumenol	*	***
p-Cymen-7-ol	*	***
**PYRAZINE**		
Pyrazine, 2,3-dimethyl-		****
Pyrazine, 2,5-dimethyl-3-(3-methylbutyl)-		***
Pyrazine, 2,6-dimethyl-		****
Pyrazine, 2-ethyl-3,5-dimethyl-		****
Pyrazine, 2-ethyl-5-methyl-	*	***
Pyrazine, 2-ethyl-6-methyl-		****
Pyrazine, 2-methyl-6-(1-propenyl)-, (E)-		****
Pyrazine, 2-methyl-6-propyl-	*	***
Pyrazine, 3,5-diethyl-2-methyl-	*	**
Pyrazine, 3-ethyl-2,5-dimethyl-		****
Pyrazine, ethyl-		****
Pyrazine, methyl-	*	****

	Two Way ANOVA	
	**Flours**	**Product**
2-Isobutyl-3-methylpyrazine	*	
**KETONES**		
2-Heptanone		***
2-Heptanone, 4-methyl-		
2-Nonanone	*	***
2-Octanone		*
3,5-Octadien-2-one (Z)		****
3,5-Octadien-2-one, (E)	***	****
5-Hepten-2-one, 6-methyl-		****
Acetophenone	*	
**OTHERS**		
2-Methoxy-4-vinylphenol		****
Styrene	*	***
2-Pentyl furan	*	***
**ESTERS**		
2-Octanol acetate	*	
Benzoic acid methyl ester	*	

** $p < 0.05$; ** $p < 0.01$; *** $p < 0.001$; **** $p < 0.0001$; empty cell = not significant (ns).*
